# Robust Estimation of the Chronological Age of Children and Adolescents Using Tooth Geometry Indicators and POD-GP

**DOI:** 10.3390/ijerph19052952

**Published:** 2022-03-03

**Authors:** Katarzyna Zaborowicz, Tomasz Garbowski, Barbara Biedziak, Maciej Zaborowicz

**Affiliations:** 1 Department of Orthodontics and Craniofacial Anomalies, Poznań University of Medical Sciences, Collegium Maius, Fredry 10, 61-701 Poznan, Poland; biedziak@ump.edu.pl; 2 Department of Biosystems Engineering, Poznan University of Life Sciences, Wojska Polskiego 50, 60-627 Poznan, Poland; tomasz.garbowski@up.poznan.pl (T.G.); maciej.zaborowicz@up.poznan.pl (M.Z.)

**Keywords:** chronological age, dental age, age assessment, digital pantomography, digital image analysis, artificial intelligence, Gaussian processes, proper orthogonal decomposition

## Abstract

Determining the chronological age of children or adolescents is becoming an extremely necessary and important issue. Correct age-assessment methods are especially important in the process of international adoption and in the case of immigrants without valid documents confirming their identity. It is well known that traditional, analog methods widely used in clinical evaluation are burdened with a high error rate and are characterized by low accuracy. On the other hand, new digital approaches appear in medicine more and more often, which allow the increase of the accuracy of these estimates, and thus equip doctors with a tool for reliable estimation of the chronological age of children and adolescents. In this study, the work on a fast and effective metamodel is continued. Metamodels have one great advantage over all other analog and quasidigital methods—if they are well trained, a priori, on a representative set of samples, then in the age-assessment phase, results are obtained in a fraction of a second and with little error (reduced to ±7.5 months). In the here-proposed method, the standard deviation for each estimate is additionally obtained, which allows the assessment of the certainty of each result. In this study, 619 pantomographic photos of 619 patients (296 girls and 323 boys) of different ages were used. In the numerical procedure, on the other hand, a metamodel based on the Proper Orthogonal Decomposition (POD) and Gaussian processes (GP) were utilized. The accuracy of the trained model was up to 95%.

## 1. Introduction

Metric age estimation is most often used in anthropology and forensics, but also for doctors in planning and evaluating treatment outcomes, for confirming the age of illegal immigrants or children from international adoptions. Incorrect classification can lead to serious consequences, so it is extremely important to develop reliable procedures for determining the metric age. Subjective, analogue techniques used to assess the development of dentition in a young patient, despite their popularity in the clinical assessment, are characterized by low accuracy. Additionally, clear discrepancies are often noticed between the chronological age and the predicted age determined by means of appropriate scientific atlases, charts and/or tables [1,2,3,4,5,6,7,8,9,10,11,12,13,14,15,16,17,18]. The differences can be significant [1,2,3] as they can reach even 36 months [3]. Another disadvantage of the currently used methods is their time-consuming nature. This is mainly due to the fact that the assessment of the development stage of tooth buds must be accurate on the basis of tables and studies by the doctors themselves.

Dental age assessment methods can be divided into (A) clinical methods, where the time of eruption of particular groups of teeth is compared, and (B) pantomographic methods, where the process of mineralization of tooth buds is assessed. During routine dental checkups, which belong to clinical methods, the presence of groups or individual teeth in a patient can be checked. This allows dentists to determine the patient’s dental age. The advantages of this method include the fact that it is easy to use, relatively fast and noninvasive. The main disadvantages, however, include inaccuracy, which results, among other factors, from (i) the difficulty in determining whether a tooth undergoing eruption should be classified as one that has already reached the occlusal plane, and (ii) the difficulty in determining the presence of factors disturbing the eruption process [4]. Dental age can also be determined on the basis of tables and charts from which the average eruption time is determined [5]. Currently, these methods are used to detect possible dental abnormalities and to make a preliminary estimate of the patient’s age.

The pantomographic methods include techniques based on the evaluation of the mineralization of tooth buds. These methods of dental age assessment are more accurate than those based on the use of tables and charts. In the literature, one can find many proposals that differ in the number of individual stages of tooth development, as well as their type [6,7,8,9]. There are many methods for estimating metric age from a pantomographic image, such as the Demirijan, Uberlaker, and Schour and Massler methods [6,7,8,9,11,12,13,14,15,16,17]. Most of these methods were developed in the 20th century. One of the most recent methods for assessing a patient’s age is the London Atlas, which was developed in 2009 [18].

For this reason, researchers from all over the world are looking for a method to estimate a patient’s age using the latest technological advances. 

In medicine, the latest innovations in the field of computer science are used more and more often, with particular emphasis on methods based on artificial intelligence, including metamodels, which help to improve the effectiveness of treatment and the accuracy of diagnosing various diseases [19,20]. The use of artificial neural networks, especially in the processing of medical images and information, allows the generalization of data contained even in noisy X-ray images [21,22], thus avoiding misdiagnosis and more efficient diagnostics [23]. Currently, artificial neural networks (ANN) are the core of many expert systems that support doctors in the daily management of information about patients. Neural networks also help manage data during difficult procedures such as the Da Vinci Robot [24,25].

The literature also includes studies on age assessment based on the analysis of the deterioration of the condition of the teeth [26]. The study describes the use of Cone Beam Computed Tomography (CBCT) in the assessment of tooth deterioration. Age estimation was there determined on the basis of the structural changes of the tooth. The CBCT technique used in these studies is much more precise; however, it is significantly more expensive than traditional techniques and it is not common in less-developed countries. The methodology itself is not based on metrics or indexes, nor does it use automated image-analysis methods. The method does not lead to high accuracy either, as the obtained R value was only 0.85.

At the beginning of 2021, several articles regarding the estimation of the age of children, adolescents and adults with the use of artificial intelligence appeared. For example, the work of Mauer et al. [27] presents the possibility of determining age using three-dimensional images of the knee joint. However, despite the use of algorithms based on deep learning, the quality of the model was 90% and the Mean Absolute Error (MAE) was ±6 months. An additional disadvantage of this method was its high cost and time consumption. The validity of the use of artificial neural networks is questioned in the study [28], where a population of over 3000 cases aged 4 to 40 was studied. The method presented in this paper gives better results than those presented in the previous paper. Convolutional networks and measurements on cephalometric images were used, which concern not only the teeth, but also other bone parameters. Unfortunately, despite analyzing nearly 300 images, the obtained results, although indicating a correlation, were not satisfactory.

The recent work on the assessment of the metric age of children and adolescents by Zaborowicz et al. [29] follows a clear trend of using advanced digital techniques and successively increasing the accuracy of the obtained results. The paper presents a very innovative methodology based on carefully selected 21 coefficients describing the proportions of the geometrical dimensions of the selected teeth. Measurements are carried out fully automatically using the specialized tools for the analysis of pantomographic images of the patients’ dentition. The use of artificial intelligence in the work allowed to obtain very accurate results.

The present work is an extension of the work presented by Zaborowicz [29]. New elements that are additionally included here are: (a) automatic sensitivity analysis of all 21 indices to see which of them carry more information; (b) the use of data compression techniques based on the Proper Orthogonal Decomposition (POD) [30,31,32,33], which allows not only the reduction of the size of the input vector but also the denoising of the data, thereby reducing the risk of overfitting the model; (c) the use of Gaussian processes (GP) [34,35], which allows results and their uncertainty to be obtained. This model is trained once-for-all and can later be used as a ready-made tool for in situ identification of the dental age of new patients. The only requirement is the correct parameterization of the pantomographic image of a patient from the same ethnic group for which the model was constructed, based on the precollected training data. Moreover, in the process of learning hyperparameters, a sensitivity analysis is performed, thanks to which the metamodel can be further optimized in a fully automated way. The proprietary procedures have been implemented in the Mathworks software—Matlab 2021b [36]. The effectiveness of the proposed algorithms for the amplitude vectors truncated to just 7 values reached a classification accuracy of 95%.

The most important improvement compared to the previous work is the ability to perform fully automatic prioritization of geometric indicators without the need to analyze in more detail which ones are more important and which ones are less. This is particularly important in the case of patients who do not have some of the selected features, which may disturb the operation of the algorithms estimating the child’s age. Another equally important improvement is the ability to determine the age of the child and adolescent with equally good precision, but at the same time obtaining information about the uncertainty of the measurement in the form of standard deviation.

## 2. Materials and Methods

### 2.1. Research Material—Pantomographic Photos

The research material consisted of 619 digital pantomographic photos of children and adolescents obtained from the patient base of the University Center for Dentistry and Specialist Medicine in Poznan. As tooth development is most visible between the ages of 4 and 18, 296 photos of girls and 323 photos of boys in this age interval were included in the research group. Photographs showing developmental disorders and abnormalities, such as changes in the face, diseases of the hard tissues of teeth and pulp, systemic diseases or developmental defects of the face and teeth, were excluded from the study. All patients were citizens of Poland. The study did not have the characteristics of a medical experiment, therefore the Bioethics Committee of the Medical University of Poznan agreed to use them in this study.

**Figure 1:**
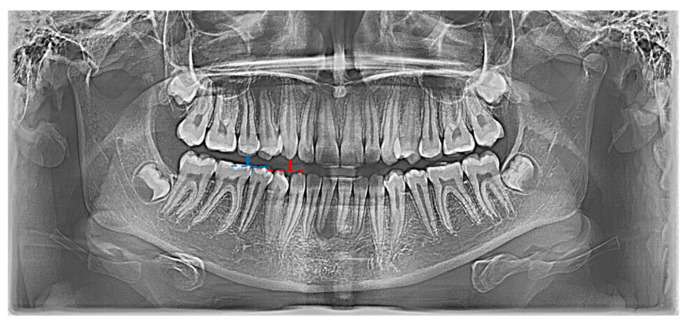
Graphical representation of indicator X01 (red |C13C43|; blue |C15C45|).

**Figure 2:**
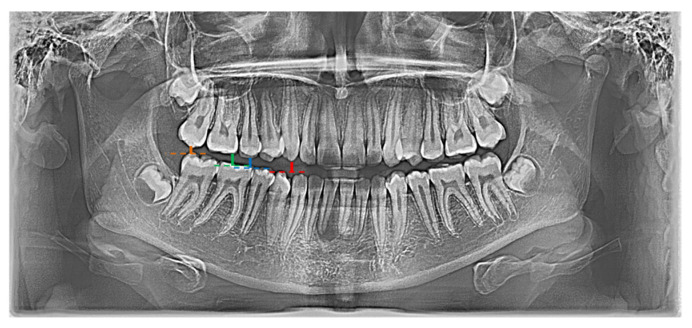
Graphical representation of selected indicators: X01 (red |C13C43|; blue |C15C45|), X02 (red |C13C43|; green |C16C46|), X03 (red |C13C43|; orange |C17C47|).

In the conducted research, a set of 21 indicators was used, estimated by Zaborowicz [37]. These indicators were selected to capture most of information about the condition of teeth of children and adolescents. As pantomographic images are not made on a fixed scale, all indicators are calculated as proportions of individual geometric distances, lengths of selected teeth, etc. Please refer to the latest work by Zaborowicz et al. [29], where all the details and descriptions of the indicators can be found. In this work, in order to avoid repetition, only the most important description of the methodology for calculating empirical data is given. Pantomographic photos taken with the Duerr Dental VistaPano S Ceph apparatus were used for the tests. This camera records digital images in the DI-COM 3.0 format, which is supported by the specialized software DBSWIN [38] used for the analysis of 16-bit grayscale, i.e., images dedicated to medicine, including oncology, ophthalmology, cardiology, surgery and dentistry [38]. To collect all indices, i.e., tooth and bone parameters the free, open-source software ImageJ 1.52a [39] was used. Figure 1 and Figure 2 show sample photos and measurements.

### 2.2. Proper Orthogonal Decomposition

The following training pairs were used here: target vectors $t_{i}\;\left(i=1,\ldots ,M\right)$ corresponding to the known age of the patient in months (in total M=619 patients aged between 52 to 214 months) and the training data collected in a matrix $U_{N\times M}$ in which the m-th column $u_{m}$ is a vector (snapshot) containing N geometrical indexes of the teeth (in total N=21 selected indicators) for each patient $t_{m}$.

As the differences between individual indicators result only from the variability of the parameters sought within a given range, snapshots are often correlated, i.e., they create almost parallel vectors in their N-dimensional space (N=21). In order to minimize these correlations, the POD method is employed here [40,41]. This method is based on the truncation of the information contained in the snapshot matrix $U=\left[u_{1},\ldots u_{M}\right]$. Such compression eliminates the elements of the input vector that are characterized by the least variability (i.e., the system is not sensitive to their change). The mathematical theory and computational procedures related to POD have their origins dating back to the early 20th century and are applicable in various fields [30,31,32,33,42,43].

The matrix U, as defined above, initially collected from M=619 patients, is used to construct the symmetric, positive (semi)definite matrix $D=UU^{T}$. Then, by calculating its eigenvalues $\lambda_{i}$ and the corresponding eigenvectors $\Phi_{i}$ an orthonormal matrix $A_{N\times M}$, consisting of the amplitudes $a_{m}$ of the snapshots $u_{m}$, can be described by the following relationship:

(1) $$A=\left[a_{1},\ldots ,a_{M}\right]=\Phi^{T}U.$$

The noticed correlation between vectors with geometric indices of individual patients means that many amplitudes $a_{i}$ in the new basis Φ can be neglected. In the literature, one can find mathematical evidence that the negligibility of such amplitudes can be quantified by the eigenvalues $\lambda_{i}$ of matrix D, see, e.g., [44]. By arranging eigenvalues in descending order, one can notice a specific threshold below which eigenvalues do not significantly increase the cumulative sum of all eigenvalues. So, by keeping only the few, say $\bar{N}$ largest eigenvalues, with $\bar{N}\ll N$, the approximation ${\bar{U}}_{\bar{N}\times M}$ of the snapshot matrix $U_{N\times M}$ is obtained by using the truncated basis $\Omega_{\bar{N}\times M}$ and the corresponding truncated amplitudes $X_{\bar{N}\times M}$:

(2) $$X=\Omega^{T}\bar{U},$$

In cases where the number of training pairs is very large, this procedure turns out to be computationally demanding and often time-consuming, but it is performed only once as preparatory work to generate the matrices Ω and X. After this work is done, each new snapshot $u^{*}$ with geometric indices of the teeth corresponding to the new patient $t^{*}$ (new because it was not used in the learning process) can now be determined by (2) by its truncated amplitude $\bar{N}$-vector $x\left(t^{*}\right)$.

### 2.3. Gaussian Processes

Gaussian processes can be illustrated by a linear regression (LR) model, which consists pf a linear function of the model parameters w and a nonlinear function of the input vector (i.e., truncated amplitude vector x):

(3) $$y\left(x,w\right)=\overset{M}{\underset{j=1}{\sum }}w_{j}\varphi_{j}\left(x\right),$$

where $\varphi_{j}\left(x\right)\;$ is a fixed basis functions of the input variables (e.g., polynomial or radial basis functions).

For the M given training patterns $\left(x_{m},\;t_{m}\right)$, $x_{m}$ being the input vector and $t_{m}$ the response for m=1…M, the parameters vector w of the linear model might be solved by the penalized least squares method:

(4) $$w={\left(\Theta^{T}\Theta +\alpha I\right)}^{-1}\Theta^{T}t,$$

where $\Theta_{N\times M}$ is a design matrix with elements defined as $\theta_{m}\left(x_{n}\right)$. Here, the regularization parameter α is called a hyperparameter and can be computed by using a validation set or by maximizing evidence of dataset p(t|α) with respect to α [45] within Bayesian inference.

Gaussian processes according to Bayesian theory are a double representation of a linear model [45], while the kernel function is here a GP covariance function. Therefore, the regression model leads to the decomposition of the target variable $y\left(x^{*}\right)$ which become the prediction for the new input vector $x^{*}$. Now, taking the conditional distribution p(y|t) as the Gaussian distribution, the mean can be determined by:

(5) $$\mathrm{mean}\left(x^{*}\right)=k^{T}C^{-1}t,$$

while the covariance by the following formula:

(6) $$\sigma^{2}\left(x^{*}\right)=c-k^{T}C^{-1}k,$$

where $C_{N\times M}$ is the covariance matrix:

(7) $$C\left(x,x^{\prime }\right)=k\left(x,x^{\prime }\right)+\beta^{-1}I,$$

where β is the variance of the target distribution and I is an identity matrix. The covariance matrix $C\left(x,\;x^{\prime }\right)$ identifies vectors x and $x^{\prime }$ closely adjacent in the input space, which then generate strongly correlated values of y(x) and $y\left(x^{\prime }\right)$ in the output space. Any function that will generate a specific non-negative covariance matrix can be used as a function of the covariance for any ordered set of (input) vectors $\left(x_{1},\ldots ,x_{M}\right)$, e.g., a stationary, nonisotropic squared exponential covariance function $k\left(x,x^{\prime }\right)$:

(8) $$k\left(x,x^{\prime }\right)=\nu \exp\left(-\frac{1}{2}\overset{M}{\underset{i}{\sum }}\omega_{i}{\left(x_{i}-x_{i}^{\prime }\right)}^{2}\right)+b,$$

where: $\omega_{i}$ controls a different distance measure in each *i*-th dimension; ν controls the vertical scale of the process; b represents the deviation that controls the vertical parallel shift of the Gaussian process. If $\omega_{i}$ is small, it means that it has little effect on the input data, therefore the i-th input data is scaled down. In general, hyperparameters play a very important role because they have a direct relationship to the sensitivity of the model with respect to the input parameters, and thus allow us to measure the importance of the input parameters.

Having defined the covariance function, it is possible to make predictions of the new input vectors. Before that, however, it is necessary to determine the hyperparameters

(9) $$r=\left[\nu ,\omega_{1},\ldots ,\omega_{N},b,\beta \right].$$

In order to find those parameters, one can search for the most probable set by maximizing the log likelihood function given by the following equation:


(10) $$\ln p\left(t|r\right)=\frac{1}{2}\ln\left|C\right|-\frac{1}{2}tC^{-1}t-\frac{N}{2}\ln2\pi ,$$

Using any gradient-based optimization algorithms, such as a first-order batch Levenberg–Marquardt Algorithm (LMA) or Trust Region Algorithm (TRA) [46]. In this study, TRA, which provides fast convergence, was used.

### 2.4. Metamodel—Training and Testing

First, the experimental data set was divided into training and test data. A different number of patient data was used for testing the model, ranging from 1/18 of all photos, that is, 34 random vector patterns, to 17/18 of all photos, which consists of 585 records. The remaining records were used to train the model. In the first stage, all 21 geometric indices without the POD procedure were used in order to determine the sensitivity of the model to individual indexes. In a model based on Gaussian processes, sensitivity is automatically determined in the learning process by scaling the hyperparameters. Next, the POD procedure was included, where the number of elements in the truncated amplitude vectors ranged from 1 to 21 in order to check for which value, the model achieves the highest efficiency.

It is expected that the fewer elements in the input vector x, the worse the estimate becomes due to insufficient information carried by the truncated amplitude vectors. Likewise, if there are more items, the estimation error becomes greater as the data contains more noise. Therefore, the search for the optimal truncation level in POD procedure seems to be extremely important and is an alternative to the sensitivity analysis built into the training algorithm in Gaussian processes.

## 3. Results

The result of this research is a new stochastic methodology for determining the chronological age of children aged 4 to 18 (52 to 214 months). A set of 21 tooth and bone parameters were used here. Those indicators were developed on the basis of digital pantomographic images by Zaborowicz [37]. A metamodel based on Gaussian processes was used here with and without compressing the input data with proper orthogonal decomposition. Two error measurements were used here to compare the performance of different models, namely scaled mean absolute error (%):

(11) $$\mathrm{SMAE}=100\frac{1}{N}\overset{N}{\underset{i=1}{\sum }}\left(1-\frac{y_{i}}{t_{i}}\right),$$

and mean absolute error (months):

(12) $$\mathrm{MAE}=\frac{1}{N}\overset{N}{\underset{i=1}{\sum }}\left|t_{i}-y_{i}\right|,$$

**Figure 3:**
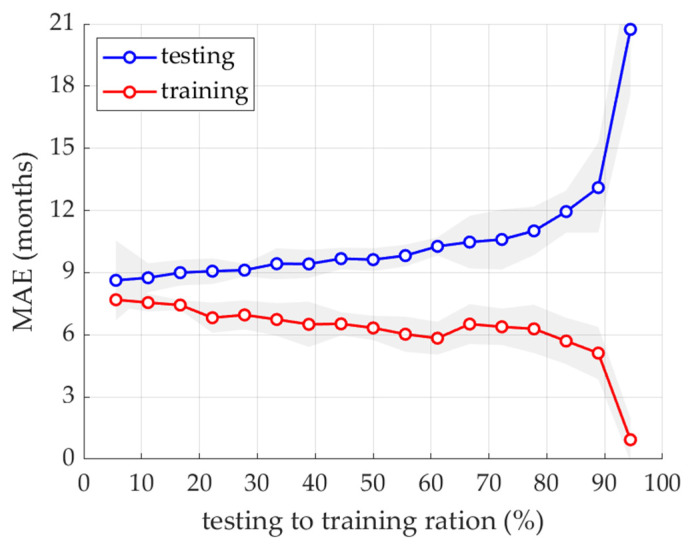
Mean absolute error of training and testing data computed by a model without POD truncation and with different number of testing and training sets.

First, a model was built without using the POD procedure. A full 21-element vector with normalization of each element was used for training. The error of the training set (MAE) ranges from 1 to 7.5 months, while the error of the test set (MAE) from 8.8 to almost 20.8 months (see Figure 3), depending on the ratio of the training set to the test set.

**Figure 4:**
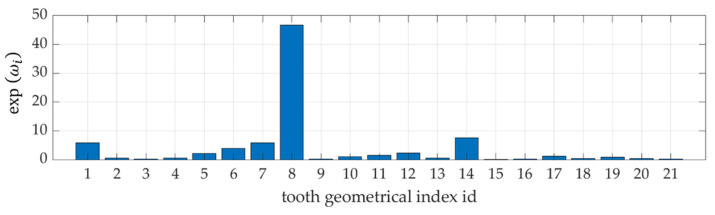
The results of sensitivity analysis for all 21 indicators.

Thanks to the built-in functionality of the training algorithm, in which the hyperparameters with the highest value point to the element of the input vector with the greatest impact on the model. The results of this specific sensitivity analysis are shown in Figure 4.

Then, a model was prepared in which the cut-off level of the amplitude vector was changed from 1 to 21 elements. Results in the form of calculated eigenvalues of matrix D, cumulative sum of eigenvalues, the SMAE and MAE values for both the training set and the testing set are shown in the Table 1. The cumulative sum was calculated using the following formula:

(13) $$r_{j}=100\overset{N^{*}}{\underset{j=1}{\sum }}\lambda_{j}\overset{N}{\underset{i=1}{\sum }}\lambda_{i}^{-1},$$

where $N^{*}$ is the cutoff level and N is the number of all eigenvalues (here N=21).

Table 1 presents the results of the estimation using models with different levels of truncation of the input vector. The results are intentionally presented separately for the training set and the test set as it is obvious that the training set will achieve a much lower MAE or SMAE value. This is especially important when these results are to be compared with the results obtained with other commercial tools, where the quality of the prediction is often given as the weighted error MAE from two sets. This is an obvious false assumption, because summing these values for large training sets that produce very small error values and a small test set that has a relatively large error, the actual fit of the model to the new patterns is completely distorted. Therefore, in the further part of this work, all results obtained by the proposed models will be presented for the test set (1/18 of all set, never used to train the model).

**Figure 5:**
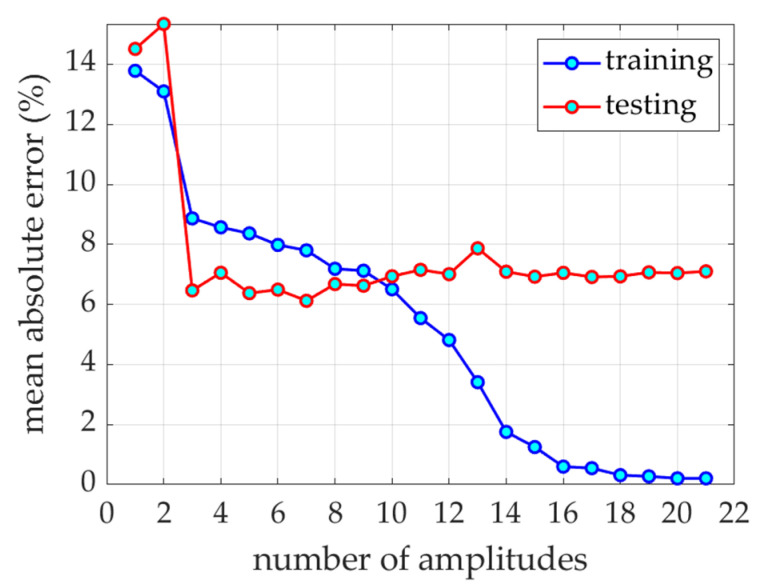
SAME for testing and training set with respect to number of input amplitudes.

Figure 5 shows the graph of the SMAE error for the training and test set depending on the number of amplitudes at the model input.

The seventh row is marked in the Table 1—in this cut-off level, the smallest SMAE and MAE error for the testing set was obtained. In the further part, the detailed values of the obtained predictions for this particular case are presented.

**Figure 6:**
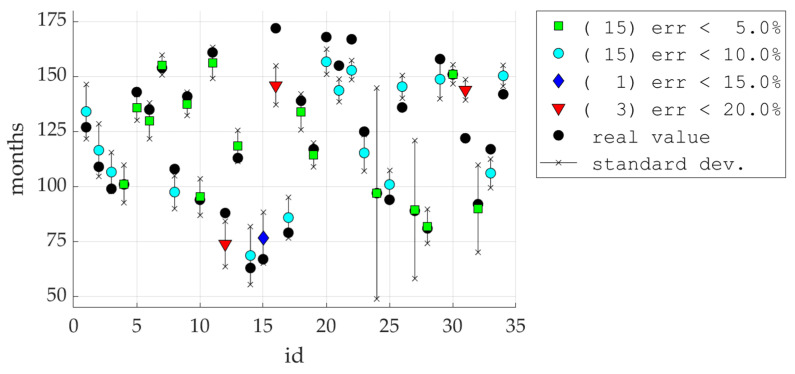
Differences between the values predicted by the 7-amplitude model and the actual values.

**Figure 7:**
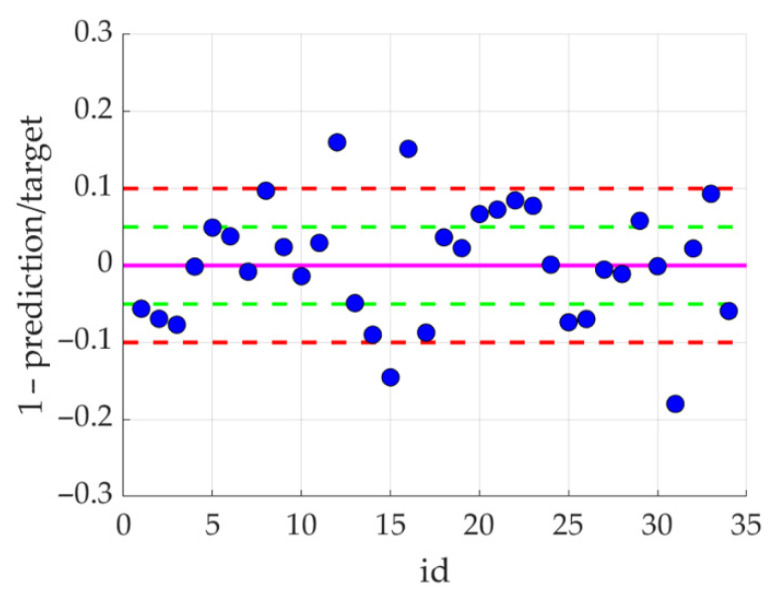
Differences between the values predicted by the 7-amplitude model and the actual values with respect to reference values (targets).

Figure 6 and Figure 7 show the detailed results obtained with the model in which the amplitude vector was cut to 7 elements.

A statistical measure of the quality of the model may be the coefficient of determination—R square—which can be determined using the formula:

(14) $$R^{2}=1-\overset{M}{\underset{i=1}{\sum }}{\left(t_{i}-y_{i}\right)}^{2}\overset{M}{\underset{i=1}{\sum }}{\left(y_{i}-\bar{y}\right)}^{-2},$$

where: M is number of targets, $t_{i}$ is a target, $y_{i}$ is a model prediction, $\bar{y}$ is a mean of target vector:

(15) $$\bar{y}=\frac{1}{M}\overset{M}{\underset{i=1}{\sum }}y_{i},$$

**Figure 8:**
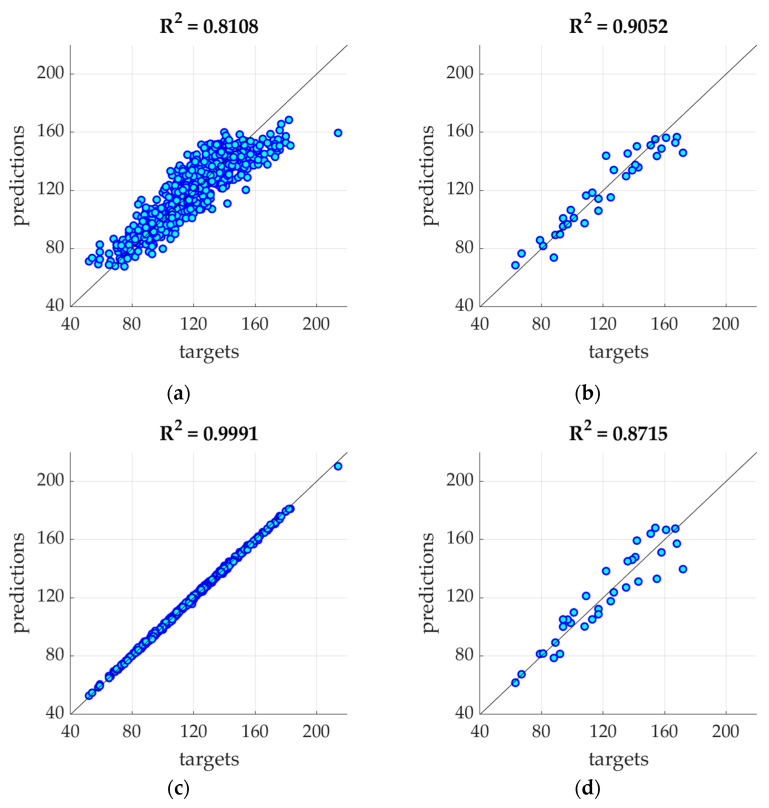
R square value for training and testing sets generated by different models: (**a**) training set—model with 7-amplitudes; (**b**) testing set—model with 7 amplitudes; (**c**) training set—model with 17 amplitudes; (**d**) testing set—model with 17 amplitudes.

Figure 8 presents the R square values for testing and training sets of the two models: (1) with 7 amplitudes; (2) with 17 amplitudes.

## 4. Discussion

From the preliminary case study presented in Figure 3, it can be concluded that the model presented here is practically independent of the ratio of the selected test set to the training set. Only when the training set is below 10% of the whole set, the results obtained with the use of the model for the test set did not exceed 12 months of the MAE error. The best results for the test set were obtained for the ratio (testing to training set) of 10%. Therefore, with more training elements, the test error decreases; however, the MSE error for the test set to the training set ratio at 10% and 90% was only between 9 and 13 months.

The first significant observation from the conducted research is the fact that the model based on all indicators but without the POD procedure is not the most optimal. It is visible in Figure 4 that the model is sensitive to only a few of the selected indicators—the greatest sensitivity of the model is to changes in the index X08, then X01, X14, X07, less to X06, X05, X12, the others have almost no impact on the sensitivity of the model. 

This observation is the basis for using POD procedures, which allow the reduction of the input vector by cutting off the least important components (amplitudes). It is worth noting that the first 7 amplitudes contain almost 99.9% of the information (see Table 1). Analyzing the SMAE generated by the model during training, it can be seen that this error systematically decreases with the increase in the number of amplitudes at the model input, but the same error for the testing set remains constant starting from three amplitudes (see Figure 5 and Table 1). This means that when training the model with a data vector containing more amplitudes, the noise is also introduced due to measurement errors, inaccuracies, etc. This can also be seen in Figure 8, where the R squared value increases when the model was trained with 17 amplitudes (with respect to model trained with 7 amplitudes), but the same model for the testing set generates a lower value coefficient of determination.

**Figure 9:**
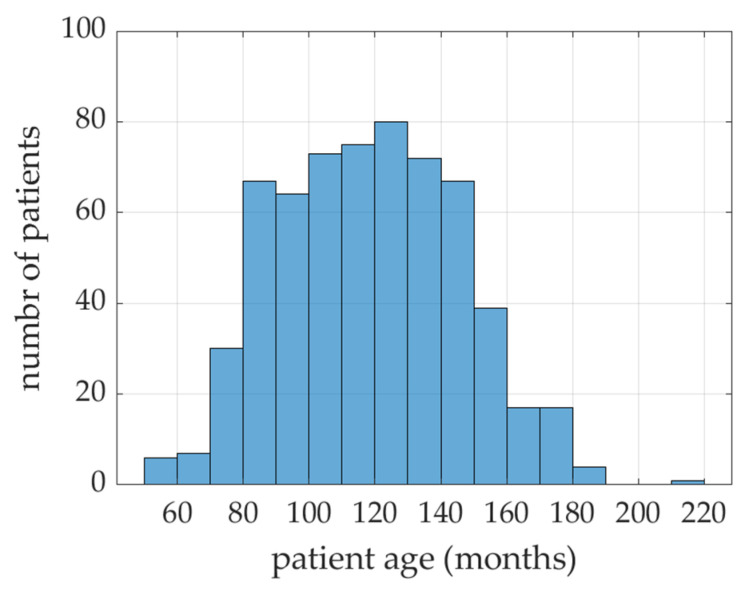
Patient age distribution.

**Figure 10:**
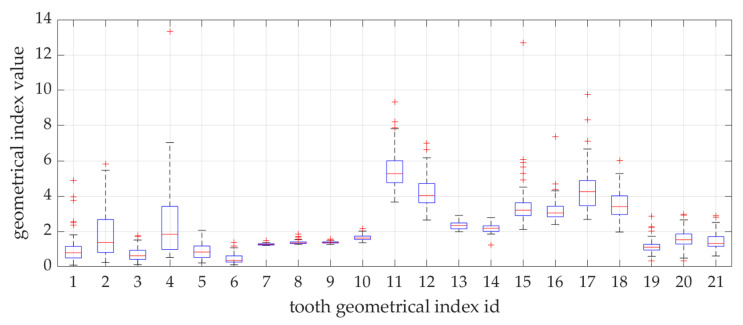
Statistical properties of the indicators X01–X21.

The results presented in Figure 6 and Figure 7 show that the model, in 30 out of 34 cases, generated results with an error of less than 10% (in 15 cases, with an error less than 5%). This is very promising, however it is puzzling why a slightly better result cannot be achieved. For a deeper analysis, a histogram was built (see Figure 9), which shows how many patients were in specific age groups—the largest number of patients is between 80–150 months. For example, for patients in the range of 120–130 months, statistical data on the variability of all indicators are summarized in Figure 10. It can be clearly seen that some parameters have very low variability (X07–X10).

Unfortunately, in other age ranges the situation is completely different—for example, the X02 parameter varies in the range from 0.235 to 464.53, similarly the X04 parameter (0.519–323.68) and the X15 parameter (0.214–43.14). This means that some of the indicators are based on the geometry of the teeth, which, for example, have not yet formed or some proportions have been incorrectly determined. Therefore, a certain increase in the accuracy of the model can be obtained, for example, by checking and, if necessary, comparing selected indicators and selecting the geometry based on their impact on the sensitivity of the model.

In order to fairly compare the results obtained with the use of the model presented in this paper with the results obtained with the use of very popular and widely used neural networks based on deep learning, only the test sets obtained by both models were selected for comparison. Deep learning network results for the same case can be found in our recent paper [47]. Since in the work by Zaborowicz et al. [47] the test set consisted of 25% of the whole set, the model presented in this work was also rebuilt so as to fairly compare the results. Ultimately, the error MAE = 8.8 ± 1.0 month was obtained, while the same error for test set obtained with the use of deep learning neural networks was MAE = 10.0 months. This observation allows to draw a general conclusion that PG-POD models can be successfully used to estimate the age of children and adolescents using large sets and are not inferior to other methods generally used in the modern world.

## 5. Conclusions

The proposed metamodel based on Gaussian processes with the input data compacting procedure using the proper orthogonal decomposition gives stable results, with mean absolute error at the level of ±7.5 months (±6.12%). Which is a significant improvement over analytical methods that can provide ±12 months at best, and a slight improvement over deep learning-based methods (±8 months). The use of POD made it possible to significantly reduce the input data—down to 7 amplitudes only—without noticeable loss of information, while maintaining only the necessary information. Once prepared (i.e., trained on collected data—panthomographic images), the model can be used to quickly assess the chronological age of children and adolescents from 4 to 18 years of age. For each new patient, the age assessment procedure is as follows: (a) a pantomographic photo is taken; (b) the geometric indices of teeth and bones are recalculated in accordance with the prepared pattern of indexes; (c) the input vector of 21 indexes is projected onto the prepared orthogonal space; (d) amplitudes are truncated to the selected level—in this case to the first 7 values; (e) the amplitude vectors are introduced into the model that generates the age of the young patient. This allowed for an easy (compared to analogue techniques) and precise assessment (up to ±7.5 month) of the dental age of a person whose age cannot or is difficult to be determined.

## Figures and Tables

**Table 1:** Results for both the analysis of the input data and the model performance. Eigenvalues of matrix D (column 2); cumulative sum of eigenvalues (column 3); mean absolute error for training and testing data (column 4–7).

Amplitude ID	Eigenvalues	Cumulative Sum of Eigenvalues	SMAE (%)		MAE (Months)	
			Training	Testing	Training	Testing
1	914,248.1	91.0098	13.7810	14.5104	14.44	14.31
2	71,644.2	98.1417	13.0938	15.3405	13.89	15.05
3	9452.9	99.0827	8.8653	6.4658	10.18	7.68
4	6619.8	99.7417	8.5683	7.0547	9.86	8.38
5	730.1	99.8144	8.3660	6.3758	9.64	7.70
6	490.9	99.8632	7.9806	6.4921	9.27	7.68
7	341.9	99.8973	7.8005	6.1207	9.07	7.53
8	323.0	99.9294	7.1876	6.6766	8.35	8.64
9	207.2	99.9500	7.1220	6.6229	8.23	8.28
10	162.4	99.9662	6.5054	6.9369	7.49	8.40
11	112.8	99.9775	5.5446	7.1527	6.37	8.74
12	76.3	99.9850	4.8145	7.0072	5.52	8.57
13	45.7	99.9896	3.4102	7.8680	3.87	10.97
14	30.3	99.9926	1.7540	7.0906	2.00	11.01
15	21.0	99.9947	1.2501	6.9232	1.42	10.43
16	17.0	99.9964	0.5975	7.0517	0.68	9.79
17	12.1	99.9976	0.5466	6.9142	0.62	9.02
18	11.2	99.9987	0.3128	6.9345	0.36	10.34
19	6.3	99.9994	0.2733	7.0676	0.31	11.99
20	4.1	99.9998	0.2075	7.0424	0.24	10.83
21	2.2	100.0000	0.2068	7.1006	0.24	15.37

## Data Availability

The study was not publicly funded. Data are not open-ended.
